# Hand Hygiene Compliance in Pediatric Emergency of a Lower-Middle Income Country: A Quality Improvement Study

**DOI:** 10.3389/fped.2022.869462

**Published:** 2022-04-29

**Authors:** Suresh Kumar Angurana, Pooja Chetal, Richa Mehta, Renu Suthar, Venkataseshan Sundaram, Ranjana Singh, Rupinder Kaur, Harinder Kaur, Manisha Biswal, Praveen Kumar, Muralidharan Jayashree

**Affiliations:** ^1^Department of Pediatrics, Advanced Pediatric Centre, Postgraduate Institute of Medical Education and Research (PGIMER), Chandigarh, India; ^2^Department of Hospital Administration, Postgraduate Institute of Medical Education and Research (PGIMER), Chandigarh, India; ^3^Department of Microbiology, Postgraduate Institute of Medical Education and Research (PGIMER), Chandigarh, India

**Keywords:** hand hygiene, neonates, quality improvement, pediatric emergency, process control, PDSA

## Abstract

**Background:**

Prospective data on hand hygiene compliance in pediatric emergency department (PED) settings is limited. We studied the impact of quality improvement measures on the overall and health care personnel wise hand hygiene compliance rates in a busy PED.

**Methods:**

The baseline hand hygiene compliance rates were audited from May–July 2018. The quality improvement interventions included various structural changes to the environment, administrative changes, education and training. During the interventions, auditing was continued for 2 months (August – September 2018). Statistical Process control charts were created.

**Results:**

We observed a significant increase in overall compliance rates from 31.8 to 53.9% (*p* < 0.001). These improvements were observed in the children (29.6 to 46.4%, *p* < 0.001) as well as neonatal area (35.7% to 59.7, *p* < 0.001) of PED as well as amongst various health care personnel and in four out of the five moments of hand hygiene.

**Conclusion:**

Hand hygiene compliance improved significantly in a busy PED of a lower middle-income country following quality improvement interventions. Such improvement was observed amongst all categories of health care personnel and different types of hand hygiene opportunities. This study demonstrates the feasibility and efficacy of simple quality improvement interventions in a challenging hospital environment.

## Introduction

Healthcare-associated infections (HCAIs) are leading cause of prolonged hospitalization, increased cost of treatment, morbidity, and mortality at a global level (1–5). Most of the pathogens responsible for HCAIs are transmitted by the hands of health care providers (HCP) and hence hand hygiene is accepted as the single most efficient strategy to reduce spread of pathogenic organisms and decrease HCAIs (6–8). Attaining optimal hand hygiene compliance (HHC) remains a challenge worldwide, more so in cases of emergency departments (EDs) (8–16). Various barriers reported for proper hand hygiene are skin irritation, inaccessible and inappropriate supplies, lack of knowledge about guidelines, forgetfulness, insufficient time, and high workload (8). Many studies have shown that quality improvement initiatives (QIIs) improve HHC among HCPs and reduce HCAIs (8, 12–15, 17–21).

The Paediatric emergency departments (PEDs) of low- and middle-income countries (LMIC’s) differ from high-income countries (HICs) in terms of space limitation, excessive patient load (often 200–300% bed occupancy rates), poor infrastructure, inadequate manpower, inadequate supplies, rapid turn-over of patients, and increased work pressure among HCPs (22, 23). All these factors contribute to poor HHC, leading to higher incidence of HCAIs, morbidity, and mortality. The rationale for the current QII stems from the fact that the pediatric emergency department (PED) of the index hospital caters to a large referral patient load of sick children and neonates, has a high turn over, and witness a significant shortage of HCPs as compared to the National and International standards. Neonates of the PED have longer stay due to shortage of specialized beds, exposing them to the overcrowded PED for unusually longer durations. Such peculiarities would increase the risk of breech in many of the infection control practices (ICPs) including HHC. Hence, we planned this study to audit the baseline HHC and to initiate QII to improve the overall compliance rates as well as compliances amongst various types of HCP’s and for various components of the “My five moments” of hand hygiene.

## Materials and Methods

This QII study was done in the PED of a tertiary care teaching hospital over 5 months (May-September 2018) after obtaining the approval from the Institute Ethics Committee (INT/IEC/2018/000788, dated 24/05/2018). Parental consent was waived off as the study did not include any intervention on the patients.

### Study Place Characteristics

The study place is a PED with two key areas with a triage zone common for both the areas. First is a 22-bedded Pediatric (post-neonatal) area with 5 beds for critically ill children. The second area is a 20-bedded neonatal unit catering to outborn neonates with 10 of them catering to the intensive care needs of sick neonates with mechanical ventilation facilities. On an average, there are around 25,000 patient visits and 11,000 patient admissions annually in the PED and at any given time point, there are ∼50 Pediatric cases and ∼60 neonatal cases, either admitted or awaiting transfer to another ward following stabilization. The doctor: patient ratio in the PED is 1:7–8 and nurse: patient ratio is 1:10. Being the only level 3 Pediatric and neonatal care center of the North-west region of the country, it caters to the needs of the five neighboring states and a lot of secondary referrals from other states also.

### Study SMART Aim

The aim of this study was to achieve an absolute improvement of 20% in the overall HHC from the baseline HHC, over a period of 3 months. A 20% improvement was chosen keeping the feasibility in mind in the planned study duration.

### Study Team and Role of Members

The team for this QII constituted of staff nursing officers, members from the hospital infection control committee (HICC), a consultant from the hospital administration, members of the clinical team of the PED and the auditing team. Audits were conducted by an audit team using a mobile phone based SpeedyAudit™ Hand Hygiene Audit App (HandyMetrics Corporation, Toronto, Ontario M2J 4R3). A subset of the main team was involved in preparation of training materials (visuals and videos) and ongoing training of the HCPs as well as the patient attendants. Two of the team members acted as team leaders to guide the process and supervise the study progress. Two senior members acted as moderators of the QII by providing performance feedback to HCPs in a confidential manner without any implication of feedback on their future academic assessments. They also supervised the adequacy and reliability of data collection by doing random checks (observing the hand hygiene opportunities, data collection, teaching sessions, data entry, and data analysis). The Hospital administration consultant also helped in training and education of HCPs during intervention phase. Microbiology and infection control consultant also helped in teaching and education as well as supervising auditors.

### Baseline (Before Intervention) Audit Phase

**FIGURE 1:**
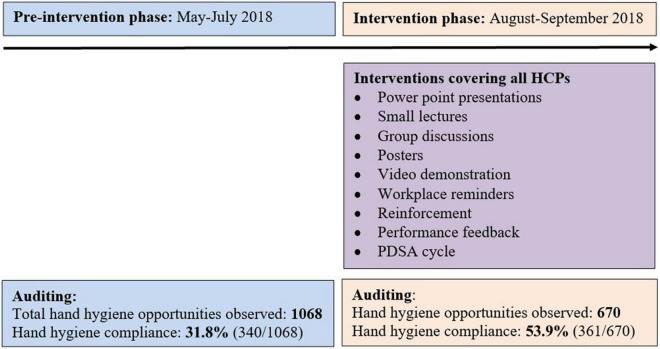
Study plan.

The baseline HHC was assessed over a duration of 12 weeks (May 2018 to July 2018) (12 data points) in the 2 clinical areas (as described above) (Figure 1). Auditing was done by direct observation in an anonymous fashion. Prior to the audits, the auditors were given knowledge about importance of hand hygiene, different opportunities of hand hygiene, methods and steps of hand hygiene, observation and assessment of the HHC, checklist for observing HHC, SpeedyAudit™ Hand Hygiene Audit App, and the components of the audit. Each observation lasted for 20 min (± 10 min) and a minimum of 10 opportunities were audited per session for the “My five moments of hand hygiene,” namely, before patient contact, before an aseptic task or procedure, after body fluid exposure, after patient contact, and after contact with patient’s environment. The HCP’s studied were physicians (staff faculty), trainee residents (MD trainee residents and senior residents), nursing officers, hospital (multipurpose helpers) and sanitation attendants (janitors) and patient attendants (parents and relatives). During the audit phase, a note was made regarding the availability of facilities for safe and effective hand washing and hand rub and these were treated as qualitative parameters. Following the baseline audit, a RUN chart was made to discuss the HHC amongst the QII team to identify and implement the interventions.

### Interventions

**FIGURE 2:**
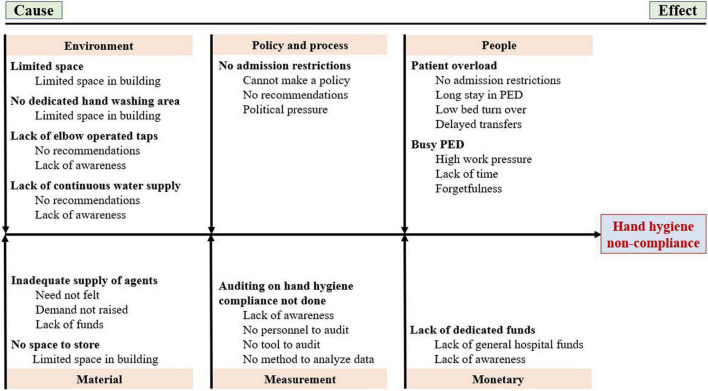
Fish bone diagram showing cause-and-effect analysis.

The intervention phase was for 8 weeks (August-September 2018) (Figure 1). Based on the results of the baseline audit and subsequent interviews, a cause-and-effect analysis diagram was drawn to classify and understand the facilitators and barriers for HHC (Figure 2). The interventions were then implemented in the PED in a plan-do-study-act (PDSA) cycle fashion.

#### Educational Interventions

Interactive teaching sessions were done as focused group discussions and short lectures on a daily basis to ensure that all HCPs were trained at least once in hand hygiene followed by reinforcement sessions done once fortnightly. A list of HCPs working in the unit was maintained and it was ensured that all HCPs working in Pediatric emergency underwent education and training sessions during intervention phase. During this phase, the auditing of HHC was continued by same auditors. Workplace reminders in the form of posters, depicting the five moments of hand hygiene and steps of hand hygiene, were displayed at prominent locations. Performance feedbacks were introduced to motivate the HCPs by displaying RUN charts to show the updated HHC rates and was given by the moderators in a confidential and non-threatening manner and the importance of hand hygiene was reinforced during those feedback activities.

#### Logistic Interventions

Dedicated hand wash corners with elbow operable taps were fitted in each area with facility for continuous supply of hot and normal water and liquid soap and sterile paper towels supplies. Alcohol-based hand rubs were placed at strategic locations in the PED. As recommended by WHO and CDC guidelines, we assumed that all HCPs should do hand hygiene with soap and water for visibly dirty hands; and alcohol-based hand rub for all other opportunities (24). Hospital administration consultant ensured the provision of supplies of liquid soap and water, sterile paper towels, and adequate alcohol-based hand rubs with dispenser throughout the study period (Table 1).

**TABLE 1:** Quality improvement interventions during the study period.

Interventions	Comments
Installation of elbow operated taps Ensuring all-weather temperature controlled continuous water supply	Required coordination of the hospital civil and construction engineering department. Inputs and recommendations from the clinicians, hospital administration and hospital infection control committee.
Dedicated and cordoned off area for hand washing	
Change from bar soaps to liquid and foam-based soap agents	
Installation of liquid soap dispensers	
Frequent feedbacks about the skin condition following frequent hand washing from the HCPs	
Making necessary changes in the soap quality (foam-based soap)	
Installation of stands for placing alcohol-based hand sanitizers at prominent places, entry, exit, and at bed sides	
Making budgetary provisions for purchase of liquid soap, hand sanitizers, and paper towels to ensure a continuous supply	
Teaching sessions and visual reinforcement methods	Group sessions, didactic lectures, focused group discussions, power point presentations, videos, and posters.
Workplace reminders	Posters displayed at prominent locations such as the entrance, near hand washing sinks, at bedsides, and the nursing stations.
Five moments of hand hygiene and the steps of hand hygiene.	
Performance feedbacks	RUN charts to show the updated HHC rates.

*HCP – health care personnel, HHC – hand hygiene compliance.*

#### Audit During Intervention Audit Phase

The HHC was assessed during the intervention phase of 8 weeks (8 data points) as well. Auditing was done by direct observation in an anonymous fashion (Figure 1). Rest of the details are same as described in baseline audit phase.

### Outcome Measures

The primary outcome measure was a process measure of overall HHC rate among HCPs, before and after intervention. Additionally, HHC was recorded for different areas, among different HCPs and patient’s attendants, and to the “my five moments” of hand hygiene. Changes in the qualitative parameters between before and after intervention were also analyzed.

### Statistical Analysis

Data was downloaded to a Microsoft Excel sheet (Microsoft Excel 2013, Microsoft, Redmond, WA, United States). The data points were further organized as numerator (complied to hand hygiene steps) and denominator (opportunity for hand hygiene) data and HHC rates were calculated and were plotted as Run charts and Statistical Process Control (SPC) charts. Data entry and statistical analysis was performed using Microsoft Excel 2013 (Microsoft, Redmond, WA, United States) and SPSS software version 21 (IBM Corp. 2012. IBM SPSS Statistics for Windows, Version 21.0. Armonk, NY, United States: IBM Corp). The charts were derived using R statistical program software with an appropriate statistical package for analyzing quality improvement data (25). From the Run charts and SPC charts (‘C’ chart for count data and ‘U’ chart for count rates), special cause (non-random) variations were separated from random (common cause) variations using the Western Electric (WE) rules as well as Anhoj rules (26–28). ‘Shift signal’ and ‘crossings signal’ were calculated for unusually long runs and unusual small number of crossings, respectively to diagnose non-random variations (29, 30). Shift signal was said to be present if any run of consecutive data points on the same side was greater than the prediction limit and crossings signal was present if the number of times the graph crossed the median was less than the prediction limit. The HHC was expressed as proportions of all opportunities of hand hygiene (overall, for different areas, categories of HCPs, and components of hand hygiene moments). Comparison of HHC in two phases/periods and among different HCPs was done by Chi-Square test. *P*-value <0.05 was considered as significant.

## Results

The study plan with phases and the cause-and-effect analysis are depicted in Figures 1, 2, respectively. The hand hygiene opportunities observed during before- and after intervention phases were 1068 and 670, respectively. The baseline (before-intervention) HHC rate combined for all areas within the Pediatric emergency was 31.8% (340 HH events out of 1068 HH opportunities) (Table 2). Staff faculty (67%, 30 out of 45) demonstrated the maximum HHC rates whereas HHC rates were as low as close to one-fifth among MD trainee residents’ group, sanitary and hospital attendants’ group, and patient attendants (21, 23, and 22%, respectively) (Table 2). Amongst the five moments of hand hygiene, lowest baseline HHC rates were observed for ‘after touching patient surroundings’ moment (17.7%) closely followed by “before clean or aseptic procedure” moment (23.4%) (Table 2).

**TABLE 2:** Hand hygiene compliance rates before and after intervention.

	Before intervention (baseline)		After intervention		“p”
	Compliant/Opportunities	Compliance rate (%)	Compliant/Opportunities	Compliance rate (%)	
Overall	340/1068	31.8	361/670	53.9	<0.001
Areas in PED					
Children area of PED	162/547	29.6	137/295	46.4	<0.001
Neonatal unit in PED	186/521	35.7	224/375	59.7	<0.001
Healthcare Personnel					
Nursing officer	170/394	43.1	102/184	55.4	0.007
Pediatric trainee resident	44/230	20.9	61/153	39.9	<0.001
Senior resident	35/105	33.3	68/111	61.3	<0.001
Staff faculty	30/45	66.7	50/69	72.5	0.5
Sanitary and hospital attendants	21/93	22.6	24/48	50	0.001
Patient’s attendant	44/201	21.9	56/105	53.3	<0.001
“My five moments” of hand hygiene					
Before touching patient	170/396	42.9	112/217	51.6	0.04
Before clean or aseptic procedure	26/111	23.4	40/74	54.1	<0.001
After body fluid exposure risk	27/60	45	32/53	60.4	0.13
After touching patient	82/303	27.1	91/169	53.8	<0.001
After touching patient surroundings	35/198	17.7	86/157	54.8	<0.001

*PED – Pediatric Emergency Department.*

**FIGURE 3:**
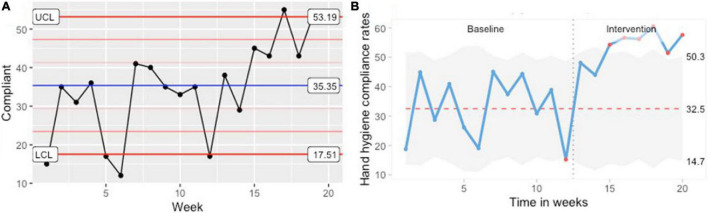
Statistical process control charts. **(A)** “C” control chart for counts (number of compliant episodes). **(B)** “U” control chart compliance rates.

The primary outcome of overall HHC rates significantly improved from 31.8% at baseline to 53.9% following the QII (*p* < 0.001). Similarly, the HHC rates in various areas within the Pediatric emergency as well amongst various types of HCP’s showed significant change after the QII (Table 2). In the HCP types, barring staff faculty stratum, rest all groups showed a significant improvement in the HHC rates (Table 2). A similar before-after improvement was observed in four out of five moments of hand hygiene (Table 2). On analyzing the trends using statistical process control charts for counts (number of compliant episodes – C chart) (Figure 3A) and count rates (U chart) (Figure 3B), the intervention phase demonstrated higher weekly HHC rates in comparison to the baseline phase with significant violation (deviation) points indicating that the variation in HHC rates observed during the intervention phase as “special cause.”

## Discussion

Ensuring hand hygiene in a busy PED of a LMIC is challenging due to the overcrowding, significant shortage of manpower, and shortage in hand hygiene resources. These limitations get further complicated by the dynamic and complex environment of an ED, rapid turn-over in the patient population, and the busy and ever pre-occupied residents and nursing officers (8). Despite evidence for the beneficial effect of hand hygiene on prevention of HCAIs (31–33), the compliance to hand hygiene in various healthcare settings including EDs has been reported to be very low, often <50% (8, 11, 12, 15, 16). In the current study, in a busy PED, we were able to demonstrate a significant increase in the overall HHC rates (20% absolute increase) following multimodal QII. Improvement was observed in both the areas of the PED (which have different patient as well as patient care activity characteristics), to four out of five moments of hand hygiene, and among all type of HCP’s and family members and attendants who took care of the admitted neonates and children.

Promoting hand hygiene among HCPs is complex and delicate due to many perceived and actual barriers. Many studies have implemented multimodal strategies of educating HCPs; making provisions for adequate water and soap supply, easily operable sinks, taps and dispensers; identifying doctor and nurse champions who will promote hand hygiene, monitoring and feedback; and creating a safety culture in the unit (8, 12–14). Kampf (20) suggested six golden rules to improve HHC: select an alcohol-based hand rub with good skin tolerability and acceptability to HCPs; ensure easy availability of hand rub; implementation of educational interventions to promote hand hygiene; creation of a budget to cover all finances relevant to preventable HCAIs; senior staff to set a good example for juniors; and ensure an appropriate patient-staff ratio. Barring the last rule, rest all were implemented to our earnest effort in the current study.

Emergency department is usually the first point of contact between patients and HCPs and majority of contacts occur here, demanding utmost HHC. Haas and Larson (34) conducted a study in an ED and demonstrated that HHC improved from 43% at baseline to 62% after intervention (introduction of wearable alcohol gel dispensers) and then dropped to 51% within 3 months after intervention. Scheithauer et al. (16) conducted a prospective triphasic before-after study (6-week observation phases interrupted by two 6-week intervention periods) in ED and demonstrated that HHC rate improved significantly from 21 to 29%, and finally to 45% (*p* < 0.001). Recently, Suo et al. (8) systematically reviewed 12 interventional studies about interventions to improve hand hygiene in ED. Authors noted that only 4 (33%) studies reported HHC of >50%. Various factors that influenced HHC included type of HCPs, indications of hand hygiene, over-crowding in ED, positive attitudes toward hand hygiene, location of the patient, hand hygiene auditing, and shift of work.

Only few studies are available in a PED setting where efforts are made to improve and sustain HHC (12–15, 21). Saint et al. (12) reported a HHC rate of 14.3% in their PED which following multimodal interventions improved significantly to 45% and was sustained at similar compliance rates after 1-year from the interventions. However, the authors reported compliance rates for the “before patient contact” moment alone. Ghazali et al. (21) conducted a study in PED among residents and nurses and documented duration and quality of hand hygiene before and after simulation-based training (SBT) and noted that SBT improved the quality and duration of hand hygiene.

We noted that the nurses had higher baseline HHC rates than the doctors, an observation made by many other reports in the past (12, 32). Moreover, pediatric trainee residents seem to form the weakest manpower link in the HHC process and ‘after touching patients surroundings’ is seemingly the weakest HHC moment. Hence, more efforts has to go in behavior modification methods to selectively address this group of HCP as well as this moment. Forming champions amongst HCP’s would be one helpful method for cross-training and cross-motivation and this can further help in sustaining the momentum achieved in HHC.

### Limitations

The current study has few important limitations. Firstly, we did not measure the sustainability of the QI measures at a later time point. Secondly, even though all attempts were made to capture enough data points across all HCP, all the locations of the PED, for all the five moments of hand hygiene and during day/night shifts and weekdays/weekends shifts, data points were inadequate for many of these subsets, which prevented us from a detailed analysis in those scenarios. Moreover, the quality of hand hygiene episodes, including diligently following all the steps of hand hygiene in a timed fashion, were not studied. Bias (Hawthorne effect) could have crept in as many of the observations were direct, which could have led to a temporary change in the behavior of the HCPs, even though the investigators attempted all through to keep the observations anonymous (35). Thirdly, we did not analyze the cost associated with structural and system changes (construction of facilities for hand washing and procurement of more hand hygiene agents) done as part of the interventions. Lastly, even though we wished to analyze the incidence rates of HCAIs as an outcome measure, we did not analyze that as part of the study for two reasons: (a) hand hygiene improvement is one of the many such interventions which when improved, would reduce the risk of HCAIs. Hence, it may not be prudent to analyze HCAI or come to a conclusion based on one piece of the jigsaw puzzle; (b) the short study duration may not be sufficient to induce changes to a major outcome like HCAI. Despite these limitations, the current study is the first of its kind to measure and report the feasibility and efficacy of a multimodal QII to improve HHC in a busy PED catering to sick and ventilated neonates and children under lots of resource limitations.

## Conclusion

To conclude, we have reported a significant improvement in the overall HHC rate in the PED from 32 to 54% following multimodal QII. This improvement was observed in the pediatric as well as sick neonatal care areas, across all HCPs, amongst families and attendants of the children, and in four out of five moments of hand hygiene. The “pediatric trainee resident” subgroup seems to be the weaker link in the chain of transmission and the “after touching the patient’s surroundings” moment seems to be the weaker moment where hand hygiene was neglected. Nevertheless, though challenging, the implementation of a QII in a busy PED was rewarding and offers scope for further improvement.

## Implications

The sustainability of this approach and its effect on key outcomes such as mortality and health care associated infections needs to be studied subsequently.

## Data Availability Statement

The raw data supporting the conclusions of this article will be made available by the authors, without undue reservation.

## Ethics Statement

The studies involving human participants were reviewed and approved by Institute Ethical Committee, PGIMER, Chandigarh (No. INT/IEC/2018/000788, dated 24/5/2018). Written informed consent for participation was not required for this study in accordance with the national legislation and the institutional requirements.

## Author Contributions

SKA: conceptualized the study, supervision of data collection, data analysis, literature review, and drafted the manuscript. PC, RM, RK, and HK: data collection. ReS: literature review and statistical analysis. VS: conceptualized the study and finalized the manuscript. RaS: material support. MB: supervised data collection and provided intellectual inputs. PK and MJ: supervised the study and provided intellectual inputs. All authors contributed to the article and approved the submitted version.

## Conflict of Interest

The authors declare that the research was conducted in the absence of any commercial or financial relationships that could be construed as a potential conflict of interest.

## Publisher’s Note

All claims expressed in this article are solely those of the authors and do not necessarily represent those of their affiliated organizations, or those of the publisher, the editors and the reviewers. Any product that may be evaluated in this article, or claim that may be made by its manufacturer, is not guaranteed or endorsed by the publisher.

## References

[1] Magill SS Edwards JR Bamberg W Beldavs ZG Dumyati G Kainer MA Multistate point-prevalence survey of health care-associated infections. *N Engl J Med.* (2014) 370:1198–208. 10.1056/NEJMoa1306801 24670166 PMC4648343

[2] Allegranzi B Pittet D. Healthcare-associated infection in developing countries: simple solutions to meet complex challenges. *Infect Control Hosp Epidemiol.* (2007) 28:1323–7. 10.1086/521656 17994510

[3] Erasmus V Daha TJ Brug H Richardus JH Behrendt MD Vos MC Systematic review of studies on compliance with hand hygiene guidelines in hospital care. *Infect Control Hosp Epidemiol.* (2010) 31:283–94. 10.1086/650451 20088678

[4] Ofek Shlomai N Rao S Patole S. Efficacy of interventions to improve hand hygiene compliance in neonatal units: a systematic review and meta-analysis. *Eur J Clin Microbiol Infect Dis.* (2015) 34:887–97. 10.1007/s10096-015-2313-1 25652605

[5] Alp E Leblebicioglu H Doganay M Voss A. Infection control practice in countries with limited resources. *Ann Clin Microbiol Antimicrob.* (2011) 10:36. 10.1186/1476-0711-10-36 22018286 PMC3225304

[6] Kampf G Kramer A. Epidemiologic background of hand hygiene and evaluation of the most important agents for scrubs and rubs. *Clin Microbiol Rev.* (2004) 17:863–93. 10.1128/CMR.17.4.863-893.2004 15489352 PMC523567

[7] Pittet D Allegranzi B Boyce J World Health Organization World Alliance for Patient Safety First Global Patient Safety Challenge Core Group of E. The world health organization guidelines on hand hygiene in health care and their consensus recommendations. *Infect Control Hosp Epidemiol.* (2009) 30:611–22. 10.1086/600379 19508124

[8] Seo HJ Sohng KY Chang SO Chaung SK Won JS Choi MJ. Interventions to improve hand hygiene compliance in emergency departments: a systematic review. *J Hosp Infect.* (2019) 102:394–406. 10.1016/j.jhin.2019.03.013 30935982

[9] Karaaslan A Kepenekli Kadayifci E Atici S Sili U Soysal A Çulha G Compliance of healthcare workers with hand hygiene practices in neonatal and pediatric intensive care units: overt observation. *Interdiscip Perspect Infect Dis.* (2014) 2014:306478. 10.1155/2014/306478 25525428 PMC4262750

[10] Pittet D Allegranzi B Sax H Dharan S Pessoa-Silva CL Donaldson L Evidence-based model for hand transmission during patient care and the role of improved practices. *Lancet Infect Dis.* (2006) 6:641–52. 10.1016/s1473-3099(06)70600-4 17008173

[11] Vikke HS Vittinghus S Giebner M Kolmos HJ Smith K Castrén M Compliance with hand hygiene in emergency medical services: an international observational study. *Emerg Med J.* (2019) 36:171–5. 10.1136/emermed-2018-207872 30692145 PMC6580871

[12] Saint S Bartoloni A Virgili G Mannelli F Fumagalli S di Martino P Marked variability in adherence to hand hygiene: a 5-unit observational study in Tuscany. *Am J Infect Control.* (2009) 37:306–10. 10.1016/j.ajic.2008.08.004 19135761

[13] Saint S Conti A Bartoloni A Virgili G Mannelli F Fumagalli S Improving healthcare worker hand hygiene adherence before patient contact: a before-and-after five-unit multimodal intervention in Tuscany. *Qual Saf Health Care.* (2009) 18:429–33. 10.1136/qshc.2009.032771 19955452

[14] di Martino P Ban KM Bartoloni A Fowler KE Saint S Mannelli F. Assessing the sustainability of hand hygiene adherence prior to patient contact in the emergency department: a 1-year postintervention evaluation. *Am J Infect Control.* (2011) 39:14–8. 10.1016/j.ajic.2010.06.015 20965610

[15] Larson EL Albrecht S O’Keefe M. Hand hygiene behavior in a pediatric emergency department and a pediatric intensive care unit: comparison of use of 2 dispenser systems. *Am J Crit Care.* (2005) 14:304–11. 10.4037/ajcc2005.14.4.304 15980421

[16] Scheithauer S Kamerseder V Petersen P Brokmann JC Lopez-Gonzalez LA Mach C Improving hand hygiene compliance in the emergency department: getting to the point. *BMC Infect Dis.* (2013) 13:367. 10.1186/1471-2334-13-367 23919402 PMC3750281

[17] Mukerji A Narciso J Moore C McGeer A Kelly E Shah V. An observational study of the hand hygiene initiative: a comparison of preintervention and postintervention outcomes. *BMJ Open.* (2013) 3:e003018. 10.1136/bmjopen-2013-003018 23793705 PMC3664348

[18] Mazi W Senok AC Al-Kahldy S Abdullah D. Implementation of the world health organization hand hygiene improvement strategy in critical care units. *Antimicrob Resist Infect Control.* (2013) 2:15. 10.1186/2047-2994-2-15 23673017 PMC3673893

[19] De la Rosa-Zamboni D Ochoa SA Laris-Gonzalez A Cruz-Córdova A Escalona-Venegas G Pérez-Avendaño G Everybody hands-on to avoid ESKAPE: effect of sustained hand hygiene compliance on healthcare-associated infections and multidrug resistance in a paediatric hospital. *J Med Microbiol.* (2018) 67:1761–71. 10.1099/jmm.0.000863 30372411

[20] Kampf G. The six golden rules to improve compliance in hand hygiene. *J Hosp Infect.* (2004) 56(Suppl. 2):S3–5. 10.1016/j.jhin.2003.12.023 15110114

[21] Ghazali AD Deilhes E Thomas J Laland C Thévenot S Richer JP Impact of a simulation-based training in hand hygiene with alcohol-based hand rub in emergency departments. *Infect Control Hosp Epidemiol.* (2018) 39:1347–52. 10.1017/ice.2018.229 30319092

[22] Obermeyer Z Abujaber S Makar M Stoll S Kayden SR Wallis LA Emergency care in 59 low- and middle-income countries: a systematic review. *Bull World Health Organ.* (2015) 93:577G–86G. 10.2471/BLT.14.148338 26478615 PMC4581659

[23] Muttalib F Gonzalez-Dambrauskas S Lee JH Steere M Agulnik A Murthy S Pediatric emergency and critical care resources and infrastructure in resource-limited settings: a multicountry survey. *Crit Care Med.* (2020) 49:671–81. 10.1097/CCM.0000000000004769 33337665

[24] Boyce JM Pittet D Healthcare Infection Control Practices Advisory Committee. Hand hygiene task F. Guideline for hand hygiene in health-care settings: recommendations of the healthcare infection control practices advisory committee and the HICPAC/SHEA/APIC/IDSA hand hygiene task force. *Infect Control Hosp Epidemiol.* (2002) 23(Suppl. 12):S3–40. 10.1086/503164 12515399

[25] R Core Team. *R: A Language and Environment for Statistical Computing.* Vienna: R Foundation for Statistical Computing (2020).

[26] Anhoj J Olesen AV. Run charts revisited: a simulation study of run chart rules for detection of non-random variation in health care processes. *PLoS One.* (2014) 9:e113825. 10.1371/journal.pone.0113825 25423037 PMC4244133

[27] Anhoj J. *qicharts2: Quality Improvement Charts. R Package Version 0.7.1.* (2020). Available online at: https://CRAN.R-project.org/package=qicharts2 (accessed January 20, 2022).

[28] Grey K. *ggQC: Quality Control Charts for ‘ggplot’. R Package Version 0.0.31.* (2018). Available online at: https://CRAN.R-project.org/package=ggQC (accessed January 20, 2022).

[29] Schilling MF. The surprising predictability of long runs. *Math Mag.* (2012) 85:141–9. 10.4169/math.mag.85.2.141

[30] Chen Z. A note on the runs test. *Model Assist Stat Appl.* (2010) 5:73–7. 10.3233/mas-2010-0142

[31] Boyce JM Pittet D Healthcare Infection Control Practices Advisory Committee, Force Hsaihht. Guideline for hand hygiene in health-care settings. recommendations of the healthcare infection control practices advisory committee and the HICPAC/SHEA/APIC/IDSA hand hygiene task force. Society for healthcare epidemiology of america/association for professionals in infection control/infectious diseases society of America. *MMWR Recomm Rep.* (2002) 51:CE1–4. 12418624

[32] Rosenthal VD Guzman S Safdar N. Reduction in nosocomial infection with improved hand hygiene in intensive care units of a tertiary care hospital in Argentina. *Am J Infect Control.* (2005) 33:392–7. 10.1016/j.ajic.2004.08.009 16153485

[33] MacDonald A Dinah F MacKenzie D Wilson A. Performance feedback of hand hygiene, using alcohol gel as the skin decontaminant, reduces the number of inpatients newly affected by MRSA and antibiotic costs. *J Hosp Infect.* (2004) 56:56–63. 10.1016/s0195-6701(03)00293-7 14706272

[34] Haas JP Larson EL. Impact of wearable alcohol gel dispensers on hand hygiene in an emergency department. *Acad Emerg Med.* (2008) 15:393–6. 10.1111/j.1553-2712.2008.00045.x 18370997

[35] Pittet D Simon A Hugonnet S Pessoa-Silva CL Sauvan V Perneger TV. Hand hygiene among physicians: performance, beliefs, and perceptions. *Ann Intern Med.* (2004) 141:1–8. 10.7326/0003-4819-141-1-200407060-00008 15238364

